# Immunoreactivity of anti-gelsolin antibodies: implications for biomarker validation

**DOI:** 10.1186/1479-5876-8-137

**Published:** 2010-12-20

**Authors:** Nicole Haverland, Gwënaël Pottiez, Jayme Wiederin, Pawel Ciborowski

**Affiliations:** 1 Department of Pharmacology and Experimental Neuroscience, University of Nebraska Medical Center, Omaha, NE 68198, USA

## Abstract

**Background:**

Proteomic-based discovery of biomarkers for disease has recently come under scrutiny for a variety of issues; one prominent issue is the lack of orthogonal validation for biomarkers following discovery. Validation by ELISA or Western blot requires the use of antibodies, which for many potential biomarkers are under-characterized and may lead to misleading or inconclusive results. Gelsolin is one such biomarker candidate in HIV-associated neurocognitive disorders.

**Methods:**

Samples from human (plasma and CSF), monkey (plasma), monocyte-derived macrophage (supernatants), and commercial gelsolin (recombinant and purified) were quantitated using Western blot assay and a variety of anti-gelsolin antibodies. Plasma and CSF was used for immunoaffinity purification of gelsolin which was identified in eight bands by tandem mass spectrometry.

**Results:**

Immunoreactivity of gelsolin within samples and between antibodies varied greatly. In several instances, multiple bands were identified (corresponding to different gelsolin forms) by one antibody, but not identified by another. Moreover, in some instances immunoreactivity depended on the source of gelsolin, e.g. plasma or CSF. Additionally, some smaller forms of gelsolin were identified by mass spectrometry but not by any antibody. Recombinant gelsolin was used as reference sample.

**Conclusions:**

Orthogonal validation using specific monoclonal or polyclonal antibodies may reject biomarker candidates from further studies based on misleading or even false quantitation of those proteins, which circulate in various forms in body fluids.

## Background

The development of global proteomic profiling in the mid-1990 s raised the expectations for quick discovery of new biomarkers [1]. More importantly, it was expected that profiling of body fluids using high throughput, sensitive and specific methods would result in bringing new and approved diagnostic and therapeutic biomarkers from bench to bedside in a fast track manner [2]. However, soon after the first large profiling experiments were performed, researchers observed several major problems: (***i***) very high dynamic range of the expression of proteins in the body fluids can reach 10^12 ^orders of magnitude, thereby excluding the possibility to quantitate both low and high abundance proteins without additional sample fractionation(s) [3]; (***ii***) range of concentration for any given protein varies from individual to individual in general population as well as in cohorts of patients; (***iii***) standard operating procedures - including sample preparation, mass spectrometers used, and bioinformatic database searching - varied between proteomic labs, resulting in variability and only partial overlap of results [4]; and (***iv***) orthogonal validation of biomarkers in body fluids is essential following discovery phase, however these methods often fail to confirm initial results [5].

Of all the issues listed above, several are beyond our control and others require more technological development; validation of quantitative proteomics data is one such issue requiring advancement [6,7]. Examples of orthogonal validation techniques for MS-based proteomics include Enzyme Linked ImmunoSorbent Assay (ELISA) [8-10] and Western blot [11,12]. In comparison, examples of parallel validation techniques include Stable Isotope Standards and Capture by Anti-Peptide Antibodies (SISCAPA) [13,14] and Multiple Reaction Monitoring (MRM) [15,16]. Each technique has advantages and drawbacks for the validation of potential biomarkers. For example, orthogonal validation using Western blot or ELISA requires the use of antibodies; some of which are not well characterized and when used, may result in misleading or skewed data.

Proteomic studies from our laboratory have shown that gelsolin is differentially expressed in the plasma and Cerebrospinal Fluid (CSF) of Human Immunodeficiency Virus (HIV)-infected individuals with and without dementia [17-19]. Likewise, gelsolin circulating in the plasma of monkeys infected with simian immunodeficiency virus (SIV) is also differentially expressed between pre-infection, acute and chronic infection [19]. We have also found that monocyte derived macrophage (MDM) activated by HIV infection *in vitro *produce and secrete gelsolin (Ciborowski, P.; Kraft-Terry, S. both unpublished). Taking this together, we postulated that if gelsolin is validated, it may become a candidate as a diagnostic biomarker and be justified to move to experiments using larger cohorts of patients. However, validation of the differential expression of gelsolin in body fluids occurred to be a challenging task, as quantitative Western blot did not confirm differential expression unambiguously. As further studies indicated this was caused by two major reasons. First, high variability in the immunoreactivity of commercially available antibodies and the variability in recognition of gelsolin originating from CSF or plasma resulted in ambiguity. Second, immunoaffinity purification of gelsolin followed by MS/MS revealed that although the gelsolin circulating in the plasma and CSF was the secreted form of gelsolin (plasma gelsolin; pGSN), several other forms in addition to the full-length molecule (86kDa) were also in circulation ranging in molecular weight from 10 kDa to 188 kDa [20]. Based on these prior studies and observations, this study focused on problems with validation of gelsolin using antibody based orthogonal assays.

## Materials and methods

### Samples and Sample Processing

Four sets of human and non-human primate samples were used throughout this project: two sets of human plasma, one set of human CSF, and one set of non-human primate plasma. Human plasma and CSF samples were previously obtained from the National NeuroAIDS Tissue Consortium (NNTC, http://www.nntc.org) under request #R101. The samples were classified based only on neurocognitive status (non-demented [ND], sub-symptomatic, or HIV-associated dementia [HAD]); no other criteria (age, race, gender, T-cell count, viral load, etc.) were applied for sample selection. Two additional sample sets were obtained: human plasma from the California NeuroAIDS Tissue Network (CNTN) at the University of California San Diego, CA (from Drs. I. Grant, R. Ellis, S. Letendre) and *Rhesus macaques *plasma samples were obtained from Dr. Robert M Donahoe, University of Utah, UT [21]. The UNMC Institutional Review Board approved the use of the human clinical samples (#196-05-EX).

Prior to any type of sample processing, proteases and virus were neutralized using a solution of 10 μL - 10% Triton X-100 and 50 μL - 20X cocktail of protease inhibitors (Sigma-Aldrich; St. Louis, MO) per mL of sample as described previously in Wiederin *et al. *[19].

Each CSF sample was split into separate parts: one was used for immunodepletion and another for immunoaffinity purification. Immunodepletion was performed as described in Rozek *et al. *[18] using the Multiple Affinity Removal Spin Cartridges Hu-6 (Agilent; Santa Clara, CA). Plasma samples from CNTN, San Diego were immunodepleted as described in Pottiez *et al. *[20] using the Seppro^® ^IgY 14 LC10 Column (Sigma-Aldrich). *Rhesus macaques *plasma samples were immunodepleted as described in Wiederin *et al. *[19] using the ProteomeLab IgY-12 High Capacity Proteome Partitioning Kit (Beckman Coulter; Fullerton, CA). Following immunodepletion, all samples were stored at -80°C.

Non-immunodepleted plasma and CSF samples from NNTC were pooled based on source and neurocognitive status immediately before immunoaffinity purification of gelsolin. A 1 mL capacity HiTrap NHS-activated HP affinity column (GE Life Sciences; Pittsburg, PA) was used for immunoaffinity purification and was performed as described in Pottiez *et al. *[20]. Protein quantity for each fraction was analyzed using a NanoDrop 2000 (ThermoScientific, Inc., Waltham, MA) and fractions containing protein were pooled, dialyzed in MilliQ water and stored at -80°C.

In addition to plasma and CSF samples, human monocytes were isolated and cultured for this project. Monocytes were isolated by leukophoresis from donors whom were HIV-1, -2, and hepatitis seronegative as described in Gendelman *et al. *[22]. These monocytes were cultured and differentiated as described in Ciborowski *et al. *[23] and infection with HIV-1_ADA _(multiplicity of infection: 0.1) occurred 7 days post-plating. Cell supernatants were collected from both HIV-infected and non-infected control cells at day 3 post-infection.

### Commercial Gelsolin and Anti-Human Gelsolin (hGSN) Antibodies

Human recombinant plasma gelsolin protein was obtained from Cytoskeleton, Inc. (Denver, CO). Human plasma gelsolin protein was obtained from Sigma-Aldrich.

Antibodies used throughout this study included mouse anti-human gelsolin (hGSN) monoclonal antibody (mAb) (BD Biosciences; San Jose, CA), goat anti-hGSN C-20 polyclonal antibody (pAb) (Santa Cruz Biotechnology, Inc.; Santa Cruz, CA), and rabbit anti-hGSN pAb (Abcam; Cambridge, MA). The appropriate horseradish peroxidase (HRP) conjugated secondary antibodies (Jackson ImmnoResearch Laboratories, Inc; West Grove, PA) were used for Western blot. A total of 1 mg goat anti-hGSN pAb antibody (Santa Cruz Biotechnology) was purified by protein-G affinity chromatography (Pierce; Rockford, IL) following manufacturer's protocol and used for immunoaffinity purification of samples.

### One dimensional gel electrophoresis (1DE) and in-gel tryptic digest

Samples were desiccated using a SpeedVac (ThermoScientific) and resolubilized in 20 μL NuPAGE (Invitrogen; Carlsbad, CA) sample buffer with reducing agent prior to heating and gel loading. 1DE was performed using NuPAGE^® ^Novex^® ^precast 4-12% Bis-Tris Gels (Invitrogen) under reducing conditions. The gel was run for 90 minutes at 100 V. Human immunoaffinity purified plasma and CSF derived gelsolin gels were fixed and stained with brilliant-blue G-colloidal concentrate (Sigma-Aldrich). Remaining samples were used for Western blot.

Bands from plasma immunoaffinity purified gelsolin samples were excised using a razor blade, destained, and digested in-gel using modified trypsin. Destaining consisted of two 30-minute washes using first 200 μL of 20 mM NH_4 _HCO_3_/50% acetonitrile (ACN), then 200 μL of 100% ACN. After destaining, the gel slices were desiccated and treated with 0.1 μg/μL sequencing grade modified trypsin (Promega; Madison, WI) overnight at 37°C. Next, the peptides were extracted using 60% ACN, 0.1% TFA solution, desiccated and resuspended in 0.1% TFA. Reverse-phase C18 Zip-Tips^® ^(Millipore; Billerica, MA) were used to purify extracted peptides following manufacturer's protocol.

### Identification by LC/ESI-MS/MS

Mass spectrometric analysis was carried out using an LC/ESI-MS/MS system in a nanospray configuration using a microcapillary reverse phase RP-C18 column (New Objectives; Woburn, MA). An LCQ-Deca XP Plus ion trap mass spectrometer (ThermoScientific, Inc.) was used to perform tandem mass spectrometry. Spectra were searched and proteins were identified following procedures stated in Pottiez *et al. *[20].

### Western blot

Following electrophoresis, proteins were transferred to a polyvinylidene fluoride (PVDF) membrane (Bio-Rad; Hercules, CA) for immunodetection as previously described in Ciborowski *et al. *[24]. Manufacturer's recommendations were followed for each antibodies used in the detection of gelsolin in samples; each primary (anti-hGSN) antibody was used at a 1:1000 dilution in phosphate buffered saline with 0.02% Tween-20 (PBST) and 10% (w/v) skim milk; each secondary antibody was used at a 1:20,000 dilution.

### ExPASy Compute pI/Mw tool

Based on the sequences identified using LC/ESI-MS/MS, the theoretical molecular weight was calculated using the ExPASy Compute pI/Mw tool http://www.Expasy.org. For each band, the peptides from the most N-terminal and C-terminal regions were selected as the form endpoints. Using the FASTA sequence for secreted pGSN, all amino acids between those N-terminal and C-terminal amino acids were identified and this shortened sequence was used to generate a theoretical molecular weight.

## Results

Previously published MS-based proteomic studies have shown that plasma gelsolin (pGSN) is differentially expressed in HIV infected humans, SIV infected monkeys and *in vitro *HIV infected MDM [18-20,23]. Prior to conducting further studies using larger cohorts of samples from patients, we attempted to validate its expression using a smaller number of samples. Our validation effort using quantitative Western blot analysis gave ambiguous results and indicated that differences in validation strongly depend on which antibody was used. Therefore, the initial goal of our study was to select an anti-gelsolin antibody that when used for quantitative Western blot analysis would most closely reflect the results of proteomic profiling.

### Specificity of anti-gelsolin antibodies

**Figure 1:**
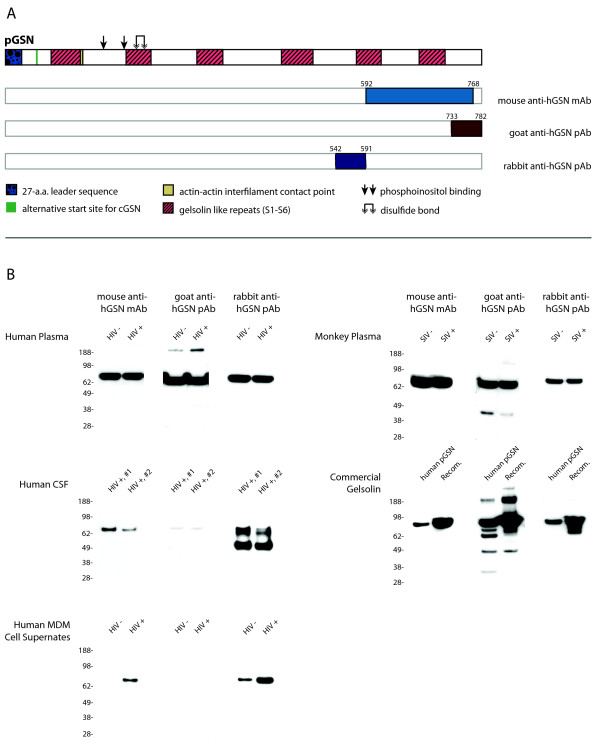
**Immunorecognition of hGSN by three antibodies in Western blot assay**. (A) The location of epitope specific to the mouse, goat and rabbit anti-hGSN antibodies are provided in reference to the full-length pGSN. Numbers above each epitope correspond to the amino acid sequence from the full-length (with signal sequence intact) pGSN containing peptides used as antigens. (B) Summary of Western blot analyses revealing that immunoreactivity of pGSN depends on not only antibodies but also source of antigen. Total protein loaded per source per lane: 25 μg of human plasma from HIV-infected individuals, 10 μg of human CSF from HIV-infected individuals, 20 μg of cell supernate from both HIV-infected and non-infected cells, 25 μg of monkey plasma from pre- and 10 days post-infection of rhesus macaques with SIV, and 2 μg each of commercially available gelsolin. Membranes from each source were probed with mouse anti-hGSN, goat anti-hGSN, and rabbit anti-hGSN (all 1:1000) and corresponding HRP-conjugated secondary antibodies (1:20,000) diluted in PBS supplemented with 10% Tween-20 and 10% (w/v) skim milk.

**Figure 2:**
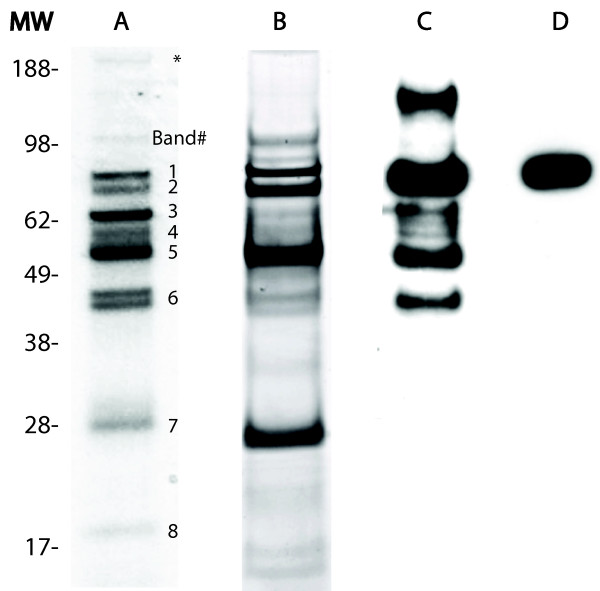
**Forms of immunoaffinity purified gelsolin**. A and B shows 1DE analysis of gelsolin immunoaffinity purified from plasma and CSF respectively. A total of 15 μg immunoaffinity purified gelsolin was loaded per lane and gels were stained with Coommasie Brilliant Blue. Eight bands (labeled in lane A) were selected for mass spectrometric identification of proteins. A total of 2 μg recombinant gelsolin was used for analysis via Western blot; banding pattern differences were seen between goat anti-hGSN (lane C) and mouse anti-hGSN (lane D). The high molecular weight band - which is identified by an asterisk - was found to contain fibronectin, a protein known to bind gelsolin. All other bands contained gelsolin, which is further discussed in Table 1.

Subsequent experiments brought to light new information concerning our previous results of Western blot validations [25] in which we observed a single band corresponding to the full-length gelsolin molecule. Concurrent experiments of immunoaffinity purification from the same samples showed multiple forms of gelsolin. This discrepancy prompted us to further explore the specificity of anti-hGSN antibodies to explain if Western blot validation of proteomic profiling might be biased and do not reflect real levels of intact and processed forms of gelsolin. From more than 20 commercially available anti-hGSN antibodies, we selected three: mouse monoclonal, goat polyclonal and rabbit polyclonal; all were raised against an epitope from the C-terminal portion of gelsolin. A sheep polyclonal anti-hGSN antibody was also tested, but results could not be obtained due to high background (data not shown). Figure 1A is a schematic diagram of the gelsolin molecule along with the location of the epitope corresponding to each antibody. Figure 1B summarizes the specificity of those three anti-hGSN antibodies used for Western blot assay against the gelsolin found various samples. It was unexpected that that monoclonal antibody raised to a synthetic peptide (located in the C-terminal end of gelsolin) reacted only with a single band of approximately 86 kDa, corresponding to the full length and intact gelsolin molecule. This indicated the conformational dependence of the antibody; moreover, it suggested that the conformation of the entire molecule - not only its C-terminal portion - is necessary for its immunoreactivity. Furthermore, Western blot analysis using either goat or rabbit pAb recognized additional forms of gelsolin with both larger and smaller molecular weights. It is likely that the smaller molecular weight forms are truncated forms of the full-length molecule based on trends observed by LC/ESI-MS/MS (Table 1). Further analysis using LC/ESI-MS/MS revealed that the larger molecular weight form (Figure 2A, asterisked band) contains fibronectin, which co-immunopurified with gelsolin; this comes as no surprise as it is well known that fibronectin binds gelsolin [26].

**Table 1:** LC/ESI-MS/MS identification of immunoaffinity purified forms of gelsolin

Gel band	M.W.*	Theoretical minimum M.W. **	Peptide sequence	Peptide position in pGSN
Band 1	86 kDa	65816.00 kDa	EVQGFESATFLGYFK	121 - 135
			HVVPNEVVVQR	151 - 161
			PALPAGTEDTAKEDAANR	251 - 268
			QTQVSVLPEGGETPLFK	347 - 363
			DPDQTDGLGLSYLSSHIANVER	371 - 392
			AGALNSNDAFVLK	558 - 570
			TPSAAYLWVGTGASEAEK	571 - 588
			AQPVQVAEGSEPDGFWEALGGK	600 - 621
			DSQEEEKTEALTSAK	687 - 701
			RYIETDPANR	702 - 711
			RTPITVVK	714 - 721

Band 2	83 kDa	50126.19 kDa	PALPAGTEDTAK	251 - 262
			QTQVSVLPEGGETPLFK	347 - 363
			DPDQTDGLGLSYLSSHIANVER.V	371 - 393
			AQPVQVAEGSEPDGFWEALGGK.A	600 - 621
			DSQEEEKTEALTSAK	687 - 701
			YIETDPANR	703 - 711

Band 3	64 kDa	64805.80 kDa	R.EVQGFESATFLGYFK.S	120 - 136
			K.PALPAGTEDTAK.E	250 - 263
			K.QTQVSVLPEGGETPLFK.Q	346 - 364
			K.DSQEEEKTEALTSAK.R	686 - 702
			R.YIETDPANR.D	702 - 712
			-.YIETDPANR.-	703 - 711

Band 4	60 kDa	49194.23 kDa	K.PALPAGTEDTAK.E	250 - 263
			R.DPDQTDGLGLSYLSSHIANVER.V	370 - 393
			K.AGALNSNDAFVLK.T	557 - 571
			R.AQPVQVAEGSEPDGFWEALGGK.A	599 - 622
			K.DSQEEEKTEALTSAK.R	686 - 702

Band 5	54 kDa	41077.02 kDa	K.QTQVSVLPEGGETPLFK.Q	346 - 364
			R.DPDQTDGLGLSYLSSHIANVER.V	370 - 393
			K.VPVDPATYGQFYGGDSYIILYNYR.H	430 - 455
			K.AGALNSNDAFVLK.T	557 - 571
			-.TGAQELLR.-	589 - 596
			R.AQPVQVAEGSEPDGFWEALGGK.A	599 - 622
			K.DSQEEEKTEALTSAK.R	686 - 702
			-.RTPITVVK.-	714 - 721

Band 6	45 kDa	36044.42 kDa	R.VPFDAATLHTSTAMAAQHGMDDDGTGQK.Q	392 - 421
			K.VPVDPATYGQFYGGDSYIILYNYR.H	430 - 455
			K.AGALNSNDAFVLK.T	557 - 571
			K.TPSAAYLWVGTGASEAEK.T	570 - 589
			R.AQPVQVAEGSEPDGFWEALGGK.A	599 - 622
			K.DSQEEEKTEALTSAK.R	686 - 702
			R.RYIETDPANR.D	701 - 712
			R.YIETDPANR.D	702 - 712
			R.RTPITVVK.Q	713 - 722

Band 7	27 kDa	30525.25 kDa	VPVDPATYGQFYGGDSYIILYNYR	431 - 454
			AGALNSNDAFVLK	558 - 570
			RYIETDPANR	702 - 711

Band 8	19 kDa	29308.93 kDa	VPVDPATYGQFYGGDSYIILYNYR	431 - 454
			DSQEEEKTEALTSAK	687 - 701

Included is the band identification (corresponding to extracted bands in Figure 1, column A), molecular weight based on electrophoretic mobility, theoretical minimum molecular weight as calculated using ExPASy Compute pI/Mw tool, identified peptides, and peptide location in secreted pGSN. The peptide -DSQEEKTEALTSAK- was the most commonly identified peptide (in 7 of 8 bands). * - Molecular weight (M.W.) is approximate based on electrophoretic mobility in 1DE SDS-PAGE. ** - Theoretical Molecular weight was approximated using the ExPASy Compute pI/Mw tool and was calculated using the first peptide position through the last peptide position as determined using MS/MS.

It was most unexpected that goat and rabbit pAb showed such great differences in immunoreactivity within samples. For example, goat pAb reacted weakly with only one form of gelsolin in the CSF whereas rabbit pAb recognized strongly two forms of gelsolin in that same sample. None of these antibodies recognized a form of gelsolin in any sample with a molecular weight lower than 45 kDa with the exception of goat pAb against commercially available human pGSN (Sigma Aldrich), which was able to recognize an additional band with a molecular weight of less than 38 kDa. In addition to the lower molecular weight forms, there were several samples in which higher molecular weight forms were detected; goat anti-hGSN pAb was able to detect these higher molecular weight forms in both human plasma and commercial gelsolin samples. Protein purification and concentration can often cause proteins to aggregate, which is a potential explanation for these higher molecular weight bands. These higher molecular weight bands warranted further investigation and using LC/ESI-MS/MS on bands excised from recombinant gelsolin, we were able to positively identify only gelsolin.

### Immunoaffinity purification of gelsolin from plasma or CSF

1DE of immunoaffinity purified CSF and plasma derived gelsolin revealed several bands with a wide range of molecular weights: approximately 17 kDa to >188 kDa (Figure 2 columns A and B). Although the relative concentration for each band varied between immunoaffinity purified gelsolin from plasma and CSF, the banding pattern remained consistent suggesting processing of gelsolin in the plasma and CSF is similar. Western blot analysis of recombinant plasma gelsolin using mouse anti-hGSN showed a single band at 86 kDa, which corresponds to the full-length gelsolin molecule (Figure 2 column D). In comparison, Western blot of this same sample using goat anti-hGSN revealed multiple bands at 166 kDa, 86 kDa, 64 kDa, 60 kDa, 54 kDa, and 45 kDa (Figure 2 column C).

Eight bands were selected for tryptic digestion and identification by LC-ESI-MS/MS from the immunoaffinity purified plasma sample (Figure 2 column A). Gelsolin was found in each band; the peptides that were identified and their exact location in secreted pGSN are included in Table 1. The approximate molecular weight for each band as estimated by electrophoretic mobility is included. Furthermore, a theoretical minimum molecular weight for each band based on the peptides identified in that band and calculated using the ExPASy Compute pI/Mw tool was also included in Table 1. Some peptides were identified in almost every band, whereas several were identified in only one or two bands. Based on the peptides identified, it was determined that the forms present in immunoaffinity purified samples were mainly truncated from the N-terminal end.

Due to the immunodetection pattern observed in the Western blots (Figure 1 and Figure 2: columns C and D), it was further postulated that the pGSN forms in bands 7 and 8 (Figure 2 column A) were likely either ***(i) ***at a concentration below the detectable threshold via Western blot, ***(ii) ***truncated at the C-terminal end and thereby lacking the immunogen required to be recognized by the antibody, and/or ***(iii) ***in an alternative conformation due to post-translational modifications or from changes occurring during 1DE and transfer.

### Immunoreactivity of gelsolin forms

**Figure 3:**
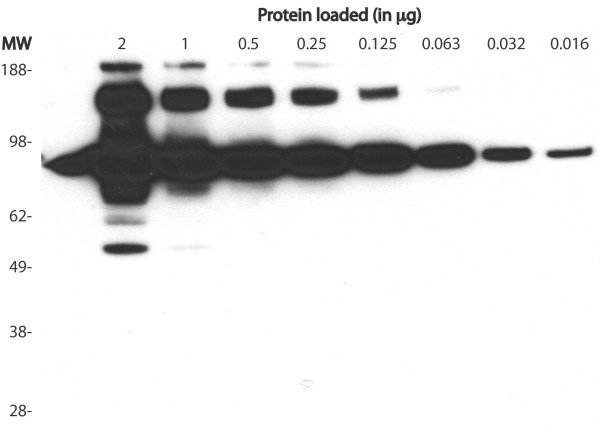
**Western blot titration of hGSN**. Titration of hGSN using Western-blot assay shows limitations of this assay in quantitation of this protein. At higher concentrations other forms than full-length molecule are detected, however, 86 kDa band representing full-length molecule is oversaturated. Conversely, at low concentration, full-length molecule can be quantitated, but the presence of all other forms is missed. Goat anti-hGSN antibody was used in this experiment.

Figure 3 includes a Western blot using goat anti-hGSN antibodies against a linear dilution of recombinant gelsolin. In the most concentrated sample (2 μg) of GSN, a total of 3 clear and distinguishable bands were detected: >188, 60 and 54 kDa both representing different forms of pGSN. Also detected were two bands at 166 kDa and 86 kDa; these bands however were not clear and distinguishable, but instead were oversaturated and unquantifiable. A 7-fold dilution (0.016 μg) of GSN resulted in only one clear, distinguishable and quantifiable band at 86 kDa. It was determined that immunodetection using goat anti-hGSN is dependent on the concentration of each form present in the sample.

**Figure 4:**
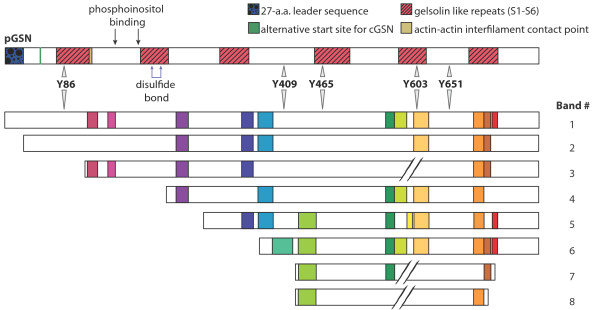
**Schematic model of full-length hGSN and proposed forms of gelsolin isolated from serum/plasma and CSF**. Band 1 represents the full-length hGSN molecule and includes its functional and structural features. This form shows electrophoretic mobility corresponding to approximately 86 kDa. hGSN was identified by tandem mass spectrometry analysis in bands 2 to 8. Based on their electrophoretic mobility and identified peptides resulting from trypsin digestion (see Table 1 for details) we estimated their approximate molecular weight and amino acid sequence coverage. Gelsolin peptides identified in each band by LC/ESI-MS/MS are colored.

Based on the banding pattern observed, peptides recognized and their location, molecular weight observed for each band and the calculated theoretical minimum molecular weight, a schematic for each band was created (Figure 4).

## Disscusion

Biomarker discovery and validation - or even the complete characterization - of the plasma and/or proximal fluids (including CSF) has been a daunting task. New biomarkers have not emerged as expected, despite the effort put forth experimentally by both small, single laboratories with limited clinical samples [3,13,27] as well as large, research organizations like the Human Plasma Proteome Project (HPPP) [28]. A conundrum has emerged with respect to validation of biomarkers following the discovery phase; is a lack of validation due to the assumptions that have been made about how a particular disease progresses or is it that the tools and reagents used are not adequate to the task? Accordingly, we postulate that a better understanding of the molecular mechanisms underlying diseases will help us to understand observed changes at the protein level and will also result in the validation of already discovered as well as new biomarker candidates.

For the majority of studies validation is based entirely or in part on immunoreactivity of specific antibodies. In terms of ELISA, this approach has been proven as very useful and accurate in many instances, e.g. measurement of bacterial proteins/toxins etc. However, when the dynamic changes of human proteins are measured or taken into consideration (including alternative splicing, post translational modification, regulated processing or degradation), immunoreactivity based assays become quite inaccurate. There has been and will continue to be attempts to improve these assays and thereby promote the acceptance of scientifically sound biomarkers. For example, Rifai *et al*. proposed a biomarker "pipeline" including discovery, qualification, verification, assay optimization, validation and commercialization to help aid in the discovery of better protein biomarkers [29]. Several of steps in this pipeline require the use of antibodies - from immunoaffinity purification in the qualification and verification stages to immunoassays like Western blot and ELISA in validation.

Experimental data that we present in this study helps to understand why in many cases validation based on immunoreactivity may lead to inconclusive or even misleading results. Moreover, we also conclude that other methods such as MRM may provide inaccurate results in the validation of biomarkers. For example, quantitative methods requiring the use of antibodies such as ELISA and quantitative Western blot will vary depending on which antibody is used (Figure 1 and Figure 2). Additionally, it was shown that the concentration of the biomarker in question might also adversely affect the results of quantitation using Western blot (Figure 3). With respect to quantitation, the different immunoaffinity purification methods used in sample preparation must also be considered. To date, our laboratory has yet to witness non-specific removal of gelsolin forms, however it has been realized that using different methods of immunoaffinity purification may adversely affect the quantitation of protein. Therefore using different methods of immunoaffinity purification should not be used in quantitative studies, but may be included in qualitative based studies. Furthermore, based on the results of this study, we reaffirmed that the conformation of the antigen does plays a key role for immunodetection; this was seen with the Western blots using mouse anti-hGSN antibody in Figures 1 and 2 being only able to detect a single gelsolin form. Additionally, goat anti-hGSN antibody was able to pull down various forms of gelsolin in human plasma during immunoaffinity purification; however it was unable to detect all the forms following 1DE, transfer and Western blot.

Our results reported here and those reported in previous papers [30-32] have a much broader implication to which method should be used for validation and eventually which potential biomarker candidate will be used or rejected from testing on larger cohorts of clinical samples.

Although ELISA has long been considered both reliable and high throughput and it is a technique that utilizes conjugated antibodies to quantify the targeted proteins, it also has limitations in its ability to differentiate between protein forms. In comparison, the Western blot - which is able to detect expression changes in the various forms of any given protein addressing the limitation of ELISA - is not a high-throughput technique and its reliability is often questioned because of saturation of chemiluminescent signal measured with X-ray films. Standardization of Western blot is much more difficult even if a fluorescently tagged secondary antibody is used. Protein microarrays, which address the issue of high-throughput, is also based on antigen-antibody interaction and must be performed using very well characterized antibodies. If an antibody used for microarrays recognizes only one or two forms, only a fragment of information about the differential expression of any protein will be received, similar to Western blot assay. Therefore, the 2D-differential in-gel electrophoresis (DIGE) profiling method - which separates full-length forms from fragments (resulting from processing or degradation) - appears to be an attractive alternative method. In our previous profiling studies using 2D-DIGE, we were able to show that the best indicator of changes of complement C3 in CSF, which is processed by multi-step well-defined mechanism, is a "residual" α-40 chain [18]. However, lack of good antibody to this fragment of C3 made orthogonal Western blot validation impossible at that time.

A novel approach known as Stable Isotope Standards and Capture by Anti-Peptide Antibodies (SISCAPA) was developed to allow for the enrichment of targeted proteins in complex samples [33] and thereby could facilitate biomarker validation. This method is based on peptide quantitation in complex mixtures such as the total tryptic digest of plasma samples. The SISCAPA method first combines immunoaffinity purified native peptide using anti-peptide antibodies immobilized on 100-nanoliter column and spiked stable-isotope-labeled internal standard peptide of the same sequence. Next, both peptides are measured by ESI-MS/MS and quantity is calculated based on the ratio of heavy (standard) to light (native) peptide, much like multiple reaction monitoring (MRM, below). Although the SISCAPA method may lead to increased sensitivity, it is a technology utilizing antibodies and therefore the same concerns with respect to ELISA and Western blot are applicable.

**Figure 5:**
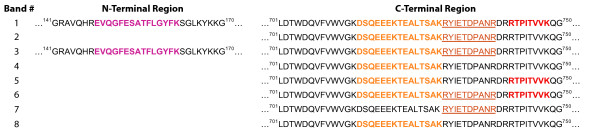
**Peptide candidates for MRM based quantitation**. Two peptides: (1) DSQEEKTEALTSAK and (2) EVQGFESATFLGYFK, derived from pGSN by trypsin digestion and representing N-terminal and C-terminal regions respectively, are well ionized and fragmented by ESI-MS/MS. As such they are excellent potential candidates for MRM based quantitation. Note that peptide (1) was identified in only 2 out of 8 bands and peptide (2) was identified in 7 out of 8 bands of pGSN circulating in plasma/CSF. Therefore, neither of these two peptides will reflect accurately levels of pGSN; additionally, use of these peptides in MRM may not validate pGSN as potential biomarker.

The MRM approach for validation is based on the comparison of abundance of selected peptides originating from a sample and spiked standard [16,34]; usually the peptides that are well ionized are selected for MRM quantitation. However, ambiguous results may occur depending on the peptide(s) chosen. For example, if one peptide is selected from N-terminal end of gelsolin and one from C-terminal region for MRM quantitation (or SISCAPA), the results of quantitative comparisons can be very different, as shown in Figure 5. More importantly, each peptide reflects a different situation; the N-terminal peptide will indicate quantity of full-length molecule and multimers whereas the C-terminal peptide will reflect the degree of gelsolin processing and/or degradation. Such an ambiguous result, if not further explained, may result in the rejection of a putative biomarker from further studies. Additionally, if a third peptide from the mid-region is selected and the quantity is averaged, the end result may not be different than the control sample and potential biomarker will also be rejected from further studies.

Gelsolin is a candidate biomarker for several neurocognitive diseases but before it can be integrated into the "biomarker pipeline" [29], further steps must be made to improve the immunoreactivity of anti-gelsolin antibodies. Without further antibody development, characterization and optimization, candidate biomarkers such as gelsolin will lack quantitative validation and thereby be unable to enter clinical assay development.

## Conclusions

Validation is one of the critical steps in bringing new biomarkers from bench to bedside in translational research. Our data presented here using gelsolin as an example, highlights a set of specific problems associated with antibody based validation methods. We also briefly describe how each of the current widely accepted methods of validation has inherent weaknesses yet each are strong enough that if used alone may lead to ambiguous or even false results. Hence, conclusions based on our experimental data have a broad application as to how we should approach validation methodologically and partially explains lack of real progress in the translation of biomarkers from bench to bedside.

## Competing interests

The authors declare that they have no competing interests.

## Authors' contributions

NH has performed the majority of the experimental work, wrote drafts of the manuscript and incorporated all suggested changes, has made drafts and final forms of all figures, and has made important contributions to the intellectual content. GP has made contributions to concept and design of the project, has been involved in revising the manuscript critically for important intellectual content, and has contributed to the designing of figures. JW has made contributions to data analysis, has been involved in revising the manuscript critically for important intellectual content, and contributed to designing of figures. PC has made substantial contributions to conception and design of the project, data analysis and interpretation, drafting of the manuscript, and has been involved in revising the manuscript critically for important intellectual content. All authors have read and approved the final manuscript.
